# Supplementary material for: A toxin-mediated policing system in Bacillus optimizes division of labor via penalizing cheater-like nonproducers
Source: eLife. 2023 Apr 25;12:e84743. doi: 10.7554/eLife.84743 (PMC10171868; doi:10.7554/eLife.84743)
Supplement: Supplementary file 1. — (a) List of strains and plasmids used in this study. (b) List of primers used in this study. [file elife-84743-supp1.docx]

**supplementary file 1a. List of strains and plasmids used in this study**

| **Strains or plasmids** | **Description** | **References or sources** |
| --- | --- | --- |
| **Strains** |  |  |
| *Escherichia coli* |  |  |
| *E. coli* Top10 | F-*mcrA*Δ (*mrr*-*hsdRMS*-*mcrBC*) ψ80*lacZ*ΔM15Δ *lacX*74 *nupG* *recA*1 *araD*139Δ (*ara-leu*) 7697 *galE*15 *galK* 16 *rpsL* (Str^R^) end A1λ- | Invitrogen (Shanghai) |
| *E. coli* BL21(DE3) | Ideal for routine T7 expression | Invitrogen (Shanghai) |
| *B. velezensis* | Wild type isolate |  |
| FZB42 | Wild type isolate | (*Chen, et al.*,*2007*) |
| SQR9 | Wild type isolate | Lab strain |
| SQR9-*P_eps_*-*gfp* | SQR9 with plasmid pNW33N-*P_eps_*-*gfp*, Cm^r^ | This study |
| SQR9-*P_tapA_*-*gfp* | SQR9 with plasmid pNW33N-*P_tapA_*-*gfp*, Cm^r^ | This study |
| SQR9-*P_bnaF_*-*gfp* | SQR9 with plasmid pNW33N-*P_bnaF_*-*gfp*, Cm^r^ | This study |
| SQR9-*P_bnaAB_*-*gfp* | SQR9 with plasmid pNW33N-*P_bnaAB_*-*gfp*, Cm^r^ | Lab strain |
| SQR9-*P_accDA_*-*gfp* | SQR9 with plasmid pNW33N-*P_accDA_*-*gfp*, Cm^r^ | This study |
| SQR9Δ*spo0A*-*P_eps_*-*gfp* | Δ*spo0A* with plasmid pNW33N-*P_eps_*-*gfp*, Cm^r^, Em^r^ | This study |
| SQR9Δ*spo0A*-*P_tapA_*-*gfp* | Δ*spo0A* with plasmid pNW33N-*P_tapA_*-*gfp*, Cm^r^, Em^r^ | This study |
| SQR9Δ*spo0A*-*P_bnaF_*-*gfp* | Δ*spo0A* with plasmid pNW33N-*P_bnaF_*-*gfp*, Cm^r^, Em^r^ | This study |
| SQR9Δ*spo0A*-*P_bnaAB_*-*gfp* | Δ*spo0A* with plasmid pNW33N-*P_bnaAB_*-*gfp*, Cm^r^, Em^r^ | This study |
| SQR9Δ*spo0A*-*P_accDA_*-*gfp* | Δ*spo0A* with plasmid pNW33N-*P_accDA_*-*gfp*, Cm^r^, Em^r^ | This study |
| SQR9Δ*spo0A*/*spo0A*-*P_eps_*-*gfp* | Δ*spo0A*/*spo0A* with plasmid pNW33N-*P_eps_*-*gfp*, Cm^r^, Em^r^, Spc^r^ | This study |
| SQR9Δ*spo0A*/*spo0A*-*P_tapA_*-*gfp* | Δ*spo0A*/*spo0A* with plasmid pNW33N-*P_tapA_*-*gfp*, Cm^r^, Em^r^, Spc^r^ | This study |
| SQR9Δ*spo0A*/*spo0A*-*P_bnaF_*-*gfp* | Δ*spo0A*/*spo0A* with plasmid pNW33N-*P_bnaF_*-*gfp*, Cm^r^, Em^r^, Spc^r^ | This study |
| SQR9Δ*spo0A*/*spo0A*-*P_bnaA_*-*gfp* | Δ*spo0A*/*spo0A* with plasmid pNW33N-*P_bnaA_*-*gfp*, Cm^r^, Em^r^, Spc^r^ | This study |
| SQR9Δ*spo0A*/*spo0A*-*P_accDA_*-*gfp* | Δ*spo0A*/*spo0A* with plasmid pNW33N-*P_accDA_*-*gfp*, Cm^r^, Em^r^, Spc^r^ | This study |
| *P_accDA_*-*mcherry* *P_bnaAB_*-*gfp* | SQR9 with plasmid pNW33N-*P_accDA_-mcherry P_bnaAB_-gfp*, Cm^r^ | This study |
| *P_eps_*-*mcherry* *P_bnaAB_*-*gfp* | SQR9 with plasmid pNW33N-*P_eps_-mcherry P_bnaAB_-gfp*, Cm^r^ | This study |
| *P_tapA_*-*mcherry* *P_bnaAB_*-*gfp* | SQR9 with plasmid pNW33N *P_tapA_*-*mcherry* *P_bnaAB_*-*gfp*, Cm^r^ | This study |
| *P_eps_*-*mcherry* *P_bnaF_*-*gfp* | SQR9 with plasmid pNW33N *P_eps_*-*mcherry* *P_bnaF_*-*gfp*, Cm^r^ | This study |
| *P_tapA_*-*mcherry* *P_bnaF_*-*gfp* | SQR9 with plasmid pNW33N- *P_tapA_-mcherry P_bnaF_-gfp*, Cm^r^ | This study |
| SQR9Δ*spo0A* | Em^r^ | Lab strain |
| SQR9Δ*spo0A*/*spo0A* | Em^r^, Spc^r^ | Lab strain |
| SQR9-*P_xyl_*-*accDA* | Replace *P_accDA_* with *P_xyl_*, Spc^r^ | This study |
| SQR9-*P_xyl_*-*accDA-P_bnaF_-gfp* | SQR9-*P_xyl_*-*accDA* with plasmid pNW33N-*P_bnaF_*-*gfp*, Cm^r^, Spc^r^ | This study |
| SQR9-*P_xyl_*-*accDA-P_bnaAB_-gfp* | SQR9-*P_xyl_*-*accDA* with plasmid pNW33N-*P_bnaAB_*-*gfp*, Cm^r^, Spc^r^ | This study |
| SQR9-*P_43_*-*bnaAB* | Replace *P_bnaAB_* with *P_43_*, Em^r^ | This study |
| SQR9Δ*bnaV* | Em^r^ | Lab strain |
| SQR9Δ*degU* | Zeocin^r^ | Lab strain |
| SQR9Δ*sinI* | Zeocin^r^ | Lab strain |
| SQR9Δ*sinR* | Zeocin^r^ | Lab strain |
| SQR9Δ*abrB* | Zeocin^r^ | Lab strain |
| SQR9Δ*comPA* | Zeocin^r^ | Lab strain |
|  |  |  |
| **Plasmids** |  |  |
| pNW33n | Cm^r^; *B.subtilis-E.coli* shuttle vector | (*Zhou, et al.*,*2018*) |
| pNW33N-*P_bnaA_*-*gfp* | pNW33N containing *P_bnaA_*-*gfp* fusion fragment | This study |
| pNW33N-*P_bnaF_*-*gfp* | pNW33N containing *P_bnaF_*-*gfp* fusion fragment | This study |
| pNW33N-*P_eps_*-*gfp* | pNW33N containing *P_eps_*-*gfp* fusion fragment | This study |
| pNW33N-*P_tapA_*-*gfp* | pNW33N containing *P_tapA_*-*gfp* fusion fragment | This study |
| pNW33N-*P_accDA_*-*gfp* | pNW33N containing *P_accDA_*-*gfp* fusion fragment | This study |
| pNW33N-*P_accDA_-mcherry P_bnaAB_-gfp* | pNW33N containing *P_bnaAB_*-*gfp* *P_accDA_*-*mcherry* fusion fragment | This study |
| pNW33N-*P_eps_-mcherry P_bnaAB_-gfp* | pNW33N containing *P_bnaAB_*-*gfp* *P_eps_*-*mcherry* fusion fragment | This study |
| pNW33N*-P_tapA_*-*mcherry* *P_bnaAB_*-*gfp* | pNW33N containing *P_bnaAB_*-*gfp* *P_tapA_*-*mcherry* fusion fragment | This study |
| pNW33N-*P_eps_*-*mcherry* *P_bnaF_*-*gfp* | pNW33N containing *P_bnaF_*-*gfp* *P_eps_*-*mcherry* fusion fragment | This study |
| pNW33N-*P_tapA_-mcherry P_bnaF_-gfp* | pNW33N containing *P_bnaF_*-*gfp* *P_tapA_*-*mcherry* fusion fragment | This study |
| pMAL-c5X-*spo0A* | pMAL-c5X containing sequence encoding Spo0A, Amp^r^ | This study |

**References**

Chen XH, Koumoutsi A, Scholz R, Eisenreich A, Schneider K, Heinemeyer I, Morgenstern B, Voss B, Hess WR, Reva O, Junge H, Voigt B, Jungblut PR, Vater J, Sussmuth R, Liesegang H, Strittmatter A, Gottschalk G, Borriss R. 2007. Comparative analysis of the complete genome sequence of the plant growth-promoting bacterium *Bacillus amyloliquefaciens* FZB42. *NAT BIOTECHNOL* **25**:1007-1014. doi:10.1038/nbt1325

Zhou X, Zhang N, Xia L, Li Q, Shao J, Shen Q, Zhang R. 2018. ResDE two-component regulatory system mediates oxygen limitation-induced biofilm formation by *Bacillus amyloliquefaciens* SQR9. *APPL ENVIRON MICROB* **84**:doi:10.1128/AEM.02744-17

**supplementary file 1b. List of primers used in this study**

| **Name** | **Sequence (5’-3’)** |
| --- | --- |
| pN-*P_tapA_-gfp*-1F | CGAGCTCGTATTGCTTGACTGCTTCG |
| pN-*P_tapA_-gfp*-1R | AGTTCTTCTCCTTTACTCATCTTTTTTTCCATTCGCAAC |
| pN-*P_tapA_-gfp*-2F | GTTGCGAATGGAAAAAAAGATGAGTAAAGGAGAAGAACT |
| pN-*P_tapA_-gfp*-2R | CCGCTCGAGTTATTTGTATAGTTCATCCAT |
| pN-*P_eps_-gfp*-1F | CGAGCTCATTGGATGGACGGATTTG |
| pN-*P_eps_-gfp*-1R | AGTTCTTCTCCTTTACTCATTAATTCTTTAAAACTCATATTC |
| pN-*P_eps_-gfp*-2F | GAATATGAGTTTTAAAGAATTAATGAGTAAAGGAGAAGAACT |
| pN-*P_bnaF_-gfp*-1F | CGAGCTCAGAGATGCATGATTTAACAT |
| pN-*P_bnaF_-gfp*-1R | AGTTCTTCTCCTTTACTCAT GAACACTTCAAAAATTTCTT |
| pN-*P_bnaF_-gfp*-2F | AAGAAATTTTTGAAGTGTTCATGAGTAAAGGAGAAGAACT |
| pN-*P_accDA_-gfp*-1F | CGAGCTCACAAGACAGCTCACGGTTAT |
| pN-*P_accDA_-gfp*-1R | AGTTCTTCTCCTTTACTCATATGATTACCTCCCTTTTGTG |
| pN-*P_accDA_-gfp*-2F | CACAAAAGGGAGGTAATCATATGAGTAAAGGAGAAGAACT |
| pN-*P_accDA_*-*mcherry*-1F | CGAGCTC ACAAGACAGCTCACGGTTAT |
| pN-*P_accDA_*-*mcherry*-1R | CTCGCCCTTGCTCACCATATGATTACCTCCCTTTTGTG |
| pN-*P_accDA_*-*mcherry*-2F | CACAAAAGGGAGGTAATCATATGGTGAGCAAGGGCGAG |
| pN-*P_accDA_*-*mcherry*-2R | CCGCTCGAGCTACTTGTACAGCTCGTCCA |
| pN-*P_bnaAB_*-*gfp* *P_eps_*-*mcherry*-1F | CGAGCTC TGAAGAATATGTTGAAACAGTT |
| pN-*P_bnaAB_*-*gfp* *P_eps_*-*mcherry*-1R | TGGACGAGCTGTACAAGTAGTTATTTGTATAGTTCATCCATGC |
| pN-*P_bnaAB_*-*gfp* *P_eps_*-*mcherry*-2F | GCATGGATGAACTATACAAATAACTACTTGTACAGCTCGTCCA |
| pN-*P_bnaAB_*-*gfp* *P_eps_*-*mcherry*-2R | CCGCTCGAG ATTGGATGGACGGATTTG |
| pN-*P_bnaF_*-*mcherry*-1F | CGAGCTCAGAGATGCATGATTTAACAT |
| pN-*P_bnaF_*-*mcherry*-1R | CTCGCCCTTGCTCACCATGAACACTTCAAAAATTTCTT |
| pN-*P_bnaF_*-*mcherry*-2F | AAGAAATTTTTGAAGTGTTCATGGTGAGCAAGGGCGAG |
| pN-*P_bnaF_*-*mcherry*-2R | CCGCTCGAGCTACTTGTACAGCTCGTCCA |
| pN-*P_bnaAB_*-*gfp* *P_tapA_*-*mcherry*-1F | CGAGCTCTGAAGAATATGTTGAAACAGTT |
| pN-*P_bnaAB_*-*gfp* *P_tapA_*-*mcherry*-1R | TGGACGAGCTGTACAAGTAGTTATTTGTATAGTTCATCCATGC |
| pN-*P_bnaAB_*-*gfp* *P_tapA_*-*mcherry*-2F | GCATGGATGAACTATACAAATAACTACTTGTACAGCTCGTCCA |
| pN-*P_bnaAB_*-*gfp* *P_tapA_*-*mcherry*-2R | CCGCTCGAGGTATTGCTTGACTGCTTCG |
| pN-*P_bnaF_*-*gfp* *P_eps_*-*mcherry*-1F | CGAGCTC AGAGATGCATGATTTAACATT |
| pN-*P_bnaF_*-*gfpP_eps_*-*mcherry*-1R | TGGACGAGCTGTACAAGTAGTTATTTGTATAGTTCATCCATGC |
| pN-*P_bnaF_*-*gfpP_eps_*-*mcherry*-2F | GCATGGATGAACTATACAAATAACTACTTGTACAGCTCGTCCA |
| pN-*P_bnaF_*-*gfpP_eps_*-*mcherry*-2R | CCGCTCGAG ATTGGATGGACGGATTTG |
| pN-*P_bnaF_*-*gfpP_tapA_*-*mcherry*-1F | CGAGCTC AGAGATGCATGATTTAACATT |
| pN-*P_bnaF_*-*gfpP_tapA_*-*mcherry*-1R | TGGACGAGCTGTACAAGTAGTTATTTGTATAGTTCATCCATGC |
| pN-*P_bnaF_*-*gfpP_tapA_*-*mcherry*-2F | GCATGGATGAACTATACAAATAACTACTTGTACAGCTCGTCCA |
| pN-*P_bnaF_*-*gfpP_tapA_*-*mcherry*-2R | CCGCTCGAG GTATTGCTTGACTGCTTCG |
| *P_xyl_*-*accDA*-UF | GAGTAGCTGAGCCTTGTAAAG |
| *P_xyl_*-*accDA*-UR | CGTTACGTTATTAGTTATCAGAACATTGTTAACCTGGT |
| *P_xyl_*-*accDA*-SpcF | ACCAGGTTAACAATGTTCTGATAACTAATAACGTAACG |
| *P_xyl_*-*accDA*-SpcR | TAAGTGTTACCCCTATAAGTTAGGTTACGTATAATGTATGCTATA |
| *P_xyl_*-*accDA*-*P_xyl_*F | TATAGCATACATTATACGTAACCTAACTTATAGGGGTAACACTTA |
| *P_xyl_*-*accDA*-*P_xyl_*R | TGTGAATATATCCTTTAACAAGTTTTCTGACTCATATCGAACATT |
| *P_xyl_*-*accDA*-BF | AATGTTCGATATGAGTCAGAAAACTTGTTAAAGGATATATTCACA |
| *P_xyl_*-*accDA*-BR | CCGTGCTTTAATAAAAAT |
| *P_43_*-*bnaAB*-UF | GCTAAGAATGGTAAAGCGTA |
| *P_43_*-*bnaAB*-UR | ACTTTTCGGGGAAATGTGTTAAACCTTATATAGTCGATAACC |
| *P_43_*-*bnaAB*-EmF | GGTTATCGACTATATAAGGTTTAACACATTTCCCCGAAAAGT |
| *P_43_*-*bnaAB*-EmR | CGAAAACATACCACCTATCATTATTTCCTCCCGTTAAATA |
| *P_43_*-*bnaAB*-*P_43_*-F | TATTTAACGGGAGGAAATAATGATAGGTGGTATGTTTTCG |
| *P_43_*-*bnaAB*-*P_43_*-R | TTCAAAATGAATTTTTCCATGTGTACATTCCTCTCTTACCTA |
| *P_43_*-*bnaAB*-BF | TAGGTAAGAGAGGAATGTACACATGGAAAAATTCATTTTGAA |
| *P_43_*-*bnaAB*-BR | TCCTTTATTAAGAAGAGCTTG |
| pMAL-spo0A-F | ACGCGTCGACGTGGAGAAAATTAAAGTTTGT |
| pMAL-spo0A-R | CGCGGATCCTTAGTGGTGGTGGTGGTGGTGCGAAGCTTTATGCTCCA |
